# Weekend sedentary behaviour and cognition three months after stroke based on the exploratory analysis of the CANVAS study

**DOI:** 10.1038/s41598-025-93149-4

**Published:** 2025-03-17

**Authors:** Natalia Egorova-Brumley, Mohamed Salah Khlif, Emilio Werden, Liam Johnson, Amy Brodtmann

**Affiliations:** 1 Melbourne School of Psychological Sciences, The University of Melbourne, Melbourne, Australia; 2 Department of Neuroscience, School of Translational Medicine, Monash University, Melbourne, Australia; 3 The Florey Institute of Neuroscience and Mental Health, Melbourne, Australia; 4 Department of Physiotherapy, Melbourne School of Health Sciences, The University of Melbourne, Melbourne, Australia; 5 School of Behavioural and Health Science, Australian Catholic University, Melbourne, Australia

**Keywords:** Stroke, Physical activity, Sedentary behavior, Cognition, Neurology, Risk factors

## Abstract

Stroke survivors experience high levels of sedentary behaviour. However, less is known about the variability in weekday-weekend patterns of sedentary behaviour and whether it is linked to cognitive performance. We examined whether there was a difference in weekend and weekday amount of time spent in sedentary and moderate-to-vigorous physical activity (MVPA) at three months post-stroke and whether there was an association between these patterns and cognitive performance at three months. We included ischaemic stroke survivors from the Cognition And Neocortical Volume After Stroke (CANVAS) cohort, with objective physical activity data estimated using the SenseWear^®^ Armband. We compared physical activity levels between 97 stroke survivors (minor severity) and 37 control participants on weekends and weekdays in sedentary and MVPA zones. We then linked these outcomes to cognitive functioning at three months. While both stroke and control groups had a comparable decrease in MVPA on weekends compared to weekdays, we observed a significant increase in sedentary activity [55 min on average (95% Confidence Interval 77 − 33) with a small effect size - partial eta squared = 0.036)] on weekends in the stroke group but not in controls. When we compared two groups of stroke participants ‘more sedentary’ vs. ‘less sedentary’—based on weekend activity, we observed a higher proportion of stroke survivors classified as cognitively impaired vs. cognitively normal in the ‘more sedentary’ group. Further analysis showed the groups differed significantly on their cognitive performance, especially in the memory domain. There is a significant difference in the amount of sedentary behaviour, but not MVPA, on weekends vs. weekdays in the stroke group. Furthermore, we demonstrate that a higher amount of sedentary activity on the weekend is associated with worse cognitive performance at three months, especially on memory tasks. These results are exploratory but suggest that decreasing sedentary behaviour, especially on the weekend, could be specifically investigated as a therapeutic target to maintain better cognition after stroke.

**Supplementary Information:**

The online version contains supplementary material available at 10.1038/s41598-025-93149-4.

## Introduction

Day of the week variability, that is – a difference in people’s behaviour between weekdays and weekends – has been demonstrated as being relevant to health outcomes. For example, fluctuations in sleep duration (when people tend to sleep more on weekends than on weekdays) has been linked to worse outcomes^1,2^. There is also evidence suggesting people experience reduced levels of physical activity on the weekend compared to weekdays^3^. Even though the distinction between weekend and weekday activity is somewhat obfuscated in older adults who are mostly retired, significant reductions in weekend activity compared to weekdays is still observed in those aged 60–75^4^. While reaching 30 min of daily moderate-to-vigorous physical activity is the goal in many stroke clinical practice guidelines, including from the American Heart and Stroke Associations, for stroke survivors to improve physical function^5^, work and family-related time pressures often necessitate alternative schedules of staying active^6^. So, in the presence of barriers to weekday activity, weekend activity can be as beneficial as weekday activity. For example, several studies demonstrated that engaging in physical exercise over the weekend, a particular pattern referred to as the ‘weekend warrior’, has been associated with positive health outcomes^6^. Understanding naturally occurring weekday-weekend patterns in activity levels after stroke could be insightful in identifying optimal targets for interventions to increase physical activity.

Staying active after stroke is very important. With documented negative effects of stroke on cognition^7–11^, engaging in regular physical activity remains one of the best ways to delay the onset of dementia and cognitive impairment^12^. Studies have described the positive effects of physical activity on cognition after stroke^13,14^. Conversely, sedentary behaviour is a known problem^15,16^ with implications for cognition^17^, although a review of several studies suggested mixed evidence^18^.

In the current exploratory study, we focused on whether there is a difference between stroke survivors and control participants in their engagement in moderate-to-vigorous physical activity (MVPA) and sedentary behaviour between weekdays and weekends, and whether any differences in activity levels are associated with cognitive outcomes at three months post-stroke.

## Methods

### Standard protocol approvals, registrations, and patient consents

The Cognition and Neocortical Volume After Stroke (CANVAS) study^7,11,19^ (Clinical Trial registration NCT02205424) recruited patients with clinically and radiologically confirmed ischaemic stroke from the stroke units at three hospitals in Melbourne, Australia: Austin Hospital, Box Hill Hospital, and the Royal Melbourne Hospital, within six weeks of stroke. Stroke severity was evaluated with the National Institutes of Health Stroke Scale (NIHSS)^12^. Ethical approval at each hospital was obtained in line with the Declaration of Helsinki and the National Health Medical Research Council (Austin Health Protocol number H2012-04650). All participants gave informed consent. The analysis presented here is exploratory and was not pre-registered in the CANVAS protocol. The primary outcomes of the CANVAS study are described elsewhere^7^.

### Participants

First-ever or recurrent ischaemic stroke patients and age- and sex-matched controls were selected from the CANVAS database. Exclusion criteria were primary haemorrhagic stroke, transient ischaemic attack, venous infarction, or history of dementia, neurodegenerative or psychiatric disorders, significant medical comorbidities, or substance abuse.

Healthy age- and sex-matched control participants with no history of cognitive impairment were recruited from a pool of community participants who had previously volunteered in MRI studies at the participating sites; spouses and age-appropriate family members of stroke participants were also approached. Inclusion and exclusion criteria were the same as for stroke patients, except for the stroke diagnosis.

We utilised all participants with physical activity data, and complete cognitive evaluations. From a total of 175 participants in the CANVAS^11^ study, 97 stroke survivors and 37 controls had complete data for the three-month time point. All available data were used for the analysis, without additional selection or inclusion criteria.

Participants were interviewed to obtain information regarding medical history, current medications, and vascular risk factors. Strokes were classified in terms of stroke etiology and stroke subtype by a stroke neurologist (AB). The NIHSS was used to assess stroke impairment and disability in the study stroke participants. Participants were screened for depression (measured with PHQ-9, with scores > 5 indicating mild depression), vascular and stroke risk factors, including type 2 diabetes mellitus (T2DM), and hypertension, Table 1. Cognitive profiles of participants were assessed as follows.

### Cognitive domains and cognitive status

A neuropsychological test battery was administered at three months post-stroke, focusing on five cognitive domains: attention, memory, visuospatial function, executive function, and language (See Supplementary Table 1 for the summary and description of the neuropsychological tests comprising each cognitive domain). We used cognitive tests validated in a stroke population with appropriate normative data, and suitable for the older participants^20^. The decision to allocate tests to each cognitive function was made by a panel comprising a stroke neurologist (AB), a clinical neuropsychologist, and two research neuropsychologists. For each cognitive task, we calculated z-scores using age-, sex-, and/or education-level-appropriate normative data. Composite z-scores for each cognitive domain were computed by averaging z-scores from respective tasks.

In addition, all participants were classified, based on their domain scores and clinical history, as: cognitively normal, cognitively impaired, or demented, as in our previous studies^11,21^.

### Physical activity data

Daytime physical activity levels were assessed at 3 months post-stroke using the SenseWear^®^ Armband (BodyMedia Inc., Pittsburgh, PA), which is a valid and reliable measurement of objective physical activity in stroke survivors^22,23^. The SenseWear^®^ Armband is a triaxial accelerometer with multi-physiological sensors, including galvanic skin resistance, heat flux, body temperature, and near-body ambient temperature. Data from these sources were combined with participants’ sex, age, height, weight, and smoking status in a proprietary algorithm to estimate minute-by-minute energy expenditure in metabolic equivalent of tasks (METs). Thresholds for classifying activity intensities as defined by the 2018 Physical Activity Guidelines Advisory Committee Scientific Report^24^ were based on multiples of resting metabolic rate (1 METs), where sedentary activity is < 1.5 METs, and MVPA is > 3.0 METs. Participants were asked to wear the SenseWear^®^ armband for ≥ 7 days and to engage in regular daily activities. A tailored pipeline developed in MATLAB was used to extract data from raw files to summarize average daytime minutes spent in sedentary and MVPA per day. Data reduction rules based on previous studies, e.g.^22^, were as follows: daytime was defined as 7:00 a.m. to 9:00 p.m. on each day and representative daily physical activity data were defined as days with ≥ 10 h of daytime wear; to ensure reliable representative daily physical activity data, we included participants with ≥ 2 days of representative daily physical activity data for weekdays and weekends. Note that physical activity data for the whole day (not only daytime) was included; the daytime thresholds were only used for selecting days with representative physical activity data, excluding days with fewer than 10 h worn. For all participants ≥ 2 days were found for weekday and weekend conditions.

### Statistical analysis

Statistical analysis was conducted using SPSS 29 (IBM, Armonk, NY, USA), with accepted levels of significance at *p* < 0.05. Collinearity was checked with variance inflation factor (VIF) and was not observed.

First, for our primary analysis, we compared the average amount of physical activity between stroke and control groups on weekend and weekdays, conducting the analysis for sedentary behaviour (< 1.5 METs) and MVPA (> 3.0 METs) separately. Given these two multiple comparisons Bonferroni-adjusted *p* < 0.025 was applied.

Next, focusing on weekend sedentary activity (as a follow up analysis to better understand the significant results from the primary analysis), we divided stroke participants into ‘more sedentary’ and ‘less sedentary’. The cut-off used to define the groups was 1080 min of < 1.5 METs (i.e., > 1080 min = more sedentary), which was equivalent to the amount of weekend sedentary activity in the control group and to the amount of activity in the overall stroke group during the weekdays. We then compared the two groups’ performance on cognitive tests in five domains by conducting separate multiway ANOVAs for each domain controlling for age, body mass index (BMI) and years of education, as these are known to affect cognitive performance. Given that 5 tests were used, Bonferroni correction (*p* < 0.01) was applied, and significant results confirmed using a correlation analysis with sedentary activity as a continuous variable. We also compared the proportion of cognitively normal to cognitively impaired individuals between the ‘more sedentary’ and ‘less sedentary’ groups, using a Chi-Square test.

## Results

Participant demographic and medical history data are summarized in Table 1.

**Table 1:** Summary of demographic and medical history data for stroke and control participants.

Variable	Stroke	Control
Age (years, SD)	69 (11)	70 (5)
Education (years, SD)	12 (3)	15 (4)
Gender* (N, % Female)	29 (30%)	14 (38%)
BMI (mean, SD)	28 (5)	26 (4)
Hypertension at baseline (N, % Yes)	60 (62%)	16 (43%)
T2DM (N, % Yes)	21 (22%)	4 (11%)
Depression PHQ-9 (mean, SD)	3.9 (4)	1.6 (2)
Admission NIHSS (Median, IQR)	2 (3)	
Admission modified rankin score (Mean, SD)	1.5 (1)^a^	
Admission montreal cognitive assessment score (Mean, SD)	23.8 (3)^b^	25.8 (2)
Stroke side		
Left (N, %)	35 (36%)	
Right (N, %)	60 (62%)	
Bilateral (N, %)	2 (2%)	
Oxfordshire classification		
LACI (N, %)	15 (16%)	
PACI (N, %)	49 (50%)	
POCI (N, %)	32 (33%)	
TACI (N, %)	1 (1%)	

Note: *all recruited participants were cisgender. ^a^only available for *N* = 65. ^b^only available for *n* = 58. *SD * standard deviation, * BMI* body-mass index, *T2DM * type 2 diabetes mellitus,*PHQ-9* Patient Health Questionnaire,*NIHSS* National Institutes of Health Stroke Scale,*IQR* interquartile range, *LACI* lacunar circulation infarction, *PACI* partial anterior circulation infarction, *POCI* posterior circulation infarction, *TACI* total anterior circulation infarction.

A comparison between stroke and control groups in weekday and weekend MVPA, controlling for age, BMI, and education, showed a significant decrease (14 min) in activity during weekends, compared to weekdays (F(1,129) = 5.139, *p* < 0.025, small effect size partial eta-squared (ηp^2^) = 0.037, the result is significant with Bonferroni correction for multiple comparisons). However, no group effects or interactions were observed (see Table 2).

A comparison between stroke and control groups in weekday and weekend sedentary behaviour showed a significant increase (32 min) in sedentary behaviour during weekends compared to weekdays (F(1,129) = 9.451, *p* = 0.003, moderate effect size ηp^2^ = 0.067, the result remains significant with Bonferroni correction for multiple comparisons) across both groups, and a trend for a group by day of the week interaction (F(1,129) = 4.937, *p* = 0.028, small effect size ηp^2^ = 0.036). A post-hoc test in the stroke group indicated that there was a 55-min increase in sedentary behaviour on the weekend, compared to weekday (F(1,129) = 10.969, *p* < 0.001, 95% Confidence Interval (CI) – 77 to – 34 min), compared to the non-significant 8-min increase in the control group. Note that repeating the analysis, with additional covariates (i.e., sex, age, BMI, depression, and education), indicated that there was a significant effect of age on the day of the week differences, but the main effect of weekday (*p* < 0.05) and a group by weekday interaction remained, albeit with lower power (F(1,122) = 3.751, *p* = 0.055, small effect size ηp^2^ = 0.03).

**Table 2:** Descriptive statistics (Mean (SD)) in minutes.

Group	*N*	Weekday MVPA	Weekend MVPA	Weekday sedentary	Weekend sedentary
Control	37	86 (54)	76 (48)	1076 (109)	1084 (128)
Stroke	97	88 (87)	70 (59)	1080 (140)	1135 (125)

*MVPA * Moderate-to-vigorous physical activity.

Focusing on weekend sedentary behaviour for the stroke group, we divided participants into those who were ‘less sedentary’ (less than 1080 min equivalent to 18 h of < 1.5 METs per day) and ‘more sedentary’ (spending more than 1080 min of < 1.5 METs). We compared the groups’ performance on cognitive tests in five domains, controlling for age, BMI, and education and observed significant group differences in the memory domain. This result survives Bonferroni correction for multiple comparisons (*p* < 0.01). The full results are presented in Table 3. Note that performing a partial correlation analysis with sedentary weekend activity as continuous variable instead of the group analysis shows a similar effect for the memory domain (*r*= – 0.26, *p* = 0.01 (2-tailed)) but not any other domains. Note also that weekend sedentary activity at 3 months did not correlate with either baseline Modified Rankin Scores (*p* = 0.495) or Montreal Cognitive Assessment (MoCA) scores (*p* = 0.208), suggesting baseline cognition and physical disability were not associated with sedentary weekend activity.

**Table 3:** Cognitive performance at 3 months on five domains (shown as z-scores) between the more sedentary and less sedentary stroke groups.

Domain	Less sedentary		More sedentary		Group difference	
	Mean	Std. Error	Mean	Std. Error	F	Sig
Memory	0.112^b^	0.153	− 0.398^b^	0.109	7.27	0.008
Attention	− 0.207^b^	0.136	− 0.423^b^	0.097	1.64	0.203
Executive	− 0.585^b^	0.177	− 0.835^b^	0.127	1.31	0.255
Visuospatial	0.262^b^	0.263	− 0.279^b^	0.190	2.76	0.100
Language	0.012^b^	0.148	0.029^b^	0.105	0.01	0.926

^b^Adjusted for Education = 12.5 years, BMI = 28, Age = 68.

Finally, we compared the distribution of cognitively normal vs. cognitively impaired stroke participants across the two groups. We found a significantly higher proportion (X(1,97) = 4.961, *p* = 0.026) of cognitively impaired individuals in the more sedentary group (46%), compared to the less sedentary group (18%), see Table 4. There were 48% left-hemisphere stroke, 34% right-hemisphere stroke and 1 bilateral stroke participants in the Cognitively Impaired More Sedentary group.

**Table 4:** Distribution of cognitive impairment in the groups by levels of sedentary behaviour.

	Cognitively impaired	Cognitively normal	Total
Less sedentary	6	27	33
More sedentary	26	38	64
Total	32	65	97

## Discussion

The current study focused on understanding differences in the amount of physical activity by disentangling MVPA and sedentary behaviour on weekends and weekdays, in stroke and control groups. We observed that, overall, there was less MVPA on weekends compared to weekdays in both the stroke and control participants. However, only the stroke group showed an increase in the amount of sedentary behaviour on the weekend. The amount of sedentary behaviour on the weekend was associated with cognitive performance, with a higher proportion of cognitive impairment at three months post-stroke and worse memory performance in the more sedentary stroke subgroup. These findings indicate that future studies should investigate whether an intervention to reduce weekend sedentary behaviour after stroke could show promise for slowing cognitive decline.

### Weekend, not weekday, activity is different in stroke vs. control groups

It is often assumed that because the weekday-weekend variability is mostly driven by work schedules^25^, it is less relevant for older adults (+ 65 years), many of whom are retired. Yet other studies that have investigated weekday-weekend patterns suggested some differences in activity levels, e.g., fewer number of steps taken during the weekend in the stroke group, although the difference was not statistically significant^26^. Our results showing differences in weekend and weekday sedentary behaviour suggest that the distinction in levels of physical inactivity exist and need to be further investigated. The gap in weekend rehabilitation compared to care during the week is often identified, with previous studies suggesting that it is feasible to conduct interventions targeting weekend activity (e.g., with a nurse), to positive effect^27^. Possibilities for online delivered training, e.g., via Zoom or recorded video sessions, could further alleviate the limitations of traditional workday hours where health professionals are available to deliver the treatment.

### Sedentary behaviour, rather than physical activity levels, differ between weekdays and weekends after stroke

Behaviourally, sedentary time and physical activity are distinct. For example, an individual can be classified as physically inactive (e.g., not meeting the physical activity and exercise recommendations for stroke survivors by the American Heart Association/American Stroke Association^5^) but spend little time in seated postures, whereas another individual can be regularly physically active (e.g., running for 30 min a day), and yet, spend prolonged periods sedentary (i.e., sitting at work)^28^.

A previous study investigating differences in weekday and weekend physical activity in stroke survivors, albeit focusing on the period of rehabilitation 44 days post-stroke, did not observe significant differences between weekday and weekend physical activity^26^. Similarly, we did not observe a difference in the MVPA levels. However, we did observe a significant difference in the amount of sedentary behaviour at three months post-stroke, suggesting that while physical activity is similar in stroke and control participants, it is being more sedentary on the weekend that sets the stroke group apart from the controls. This possibly suggests that the target could shift from increasing exercise to emphasising the need to reduce sedentary behaviour, which could be a more achievable clinical target^29–32^. Interventions to encourage more movement, e.g., increasing the number of steps appear promising, especially since sedentary behaviour reduction interventions often have shown moderate to large effects^33^, although the evidence on the efficacy of the currently available interventions is mixed^34^.

### Weekend sedentary activity and cognition

There is a link between the amount of weekend physical activity and cognition^35^. However, there is a distinction in computer-based vs. television viewing or driving type of sedentary behaviour^36^. Here, we cannot dissociate between these types. Moving during the weekend – visiting family, friends, walking, hiking – is likely to make a difference in cognitive performance. There are various interventions for reducing sedentary behaviour that can be explored, but given the findings in this study, the focus should be on their implementation specifically on the weekend, in addition to weekdays.

Engaging in more physical activity is typically associated with better overall cognitive performance, and especially in the domains of executive function and memory^37^. A specific role of physical activity on memory function was previously demonstrated in the Canadian Longitudinal Study on Aging, especially with various forms of light physical activity^38^. Consistent with this, we observed the association between sedentary activity and memory in our study. Exercise can improve memory through a variety of mechanisms, including enhancing blood circulation, synaptic plasticity, and neurogenesis, with the brain-derived neurotrophic factor cited as the most relevant mediator^39^.

### Limitations

While the study reveals an association between increased weekend sedentary behaviour and cognitive performance at three months post-stroke, it is difficult to conclude whether this is in direct response to higher sedentary behaviour or is an epiphenomenon. Causal and longitudinal studies are needed to further decipher the relationship between cognitive performance and weekend sedentary behaviour. The effects on cognition reported here are very minor, and although we did not observe significant associations between baseline cognition and sedentary behaviour, it cannot be ruled out that poorer cognition predisposes one to lead a more sedentary lifestyle rather than sedentary behaviour causing cognitive decline. It, however, does bring the question of whether increased attention to weekend sedentary behaviour after stroke to slow down cognitive decline needs to be investigated further. Future work could focus on understanding how motivation affects activity levels, and control other characteristics, e.g., stroke laterality (that was imbalanced in the current study with more right-hemisphere strokes) that could influence cognitive and physical activity levels.

### Summary

While there appears to be little difference between weekend and weekday moderate-to-vigorous physical activity between stroke or control groups, there is a significant increase in weekend sedentary behaviour in the stroke group only, suggesting that there is a meaningful distinction in weekend-weekday activity between stroke and control groups, making weekend sedentary behaviour an important subject of future research to explore the possibility that decreasing sedentary behaviour in this population might specifically benefit cognition.

## Electronic supplementary material

Below is the link to the electronic supplementary material.

Supplementary Material 1

## Data Availability

The data that support the findings of this study are available on reasonable request from the corresponding authors. All requests for raw and analysed data will be reviewed by the CANVAS investigators to determine whether the request is subject to any intellectual property or confidentiality obligations.
