# Supplementary material for: Weekend sedentary behaviour and cognition three months after stroke based on the exploratory analysis of the CANVAS study
Source: Sci Rep. 2025 Mar 17;15:9085. doi: 10.1038/s41598-025-93149-4 (PMC11914211; doi:10.1038/s41598-025-93149-4)
Supplement: Supplementary file 1 — Supplementary Material 1 [file 41598_2025_93149_MOESM1_ESM.docx]

**Supplementary Materials**

**Supplementary Table 1.** Summary of neuropsychological tests comprising each cognitive domain/function.

**Supplementary Table I.** Summary of Neuropsychological Tests Comprising Each Cognitive Domain.

| Domain | Task Name | Brief Description of Task | Index |
| --- | --- | --- | --- |
| Attention/Processing Speed | Digit Span Task (Forward and Backward) from the Wechsler Adult Intelligence Scale III (WAIS-III) [1] | The Digit Span Task involves presenting participants with a series of random digits (from 1 to 9) after which participants are required to verbally repeat the list in the same order (Forward Digit Span), or in the reverse order (Backward Digit Span). | Total number correct |
|  | Trail-Making Test [2] - *Part A* | The Trail-Making Test is a paper-pencil measure comprising 25 circles distributed over a sheet of paper. In Part A, the circles are numbered 1-25 and the participant is required to draw lines to connect the numbers in the ascending order. | Time taken (sec) |
|  | Digit-Symbol Task from the WAIS-III [1] | The Digit-Symbol Task is a paper-pencil measure requiring participants to refer to a key at the top of the page containing digits (1-9) and their corresponding symbols. Participants are required to copy the appropriate symbol in a box underneath a digit as fast as they can in the allocated time of 2-min. | Total number correct |
|  | CogState Battery [3]  *Detection Task* | A computerized simple reaction time paradigm requiring participants to press a key every time a card turns over. | Psychomotor reaction time (ms) |
|  | CogState Battery [3]  *Identification Task* | A computerized choice reaction time paradigm requiring participants to press a key in response to seeing a certain card. | Psychomotor reaction time (ms) |
|  | CogState Battery [3]  *1-Back Task* | A 1-back paradigm requiring participants to press a key every time the current image (i.e., the card) was the same as the card presented immediately prior. | Accuracy (%) |
| Memory | Hopkins-Verbal Learning Test (HVLT)-Revised [4,5] | The HVLT test consists of a 12-item word list, composed of 4 words from each of 3 semantic categories. The examiner reads each word out loud to participants at a rate of ~2-sec per word. The participant is then required to verbally recall the words in any order. The same procedure is repeated for two more trials.  After a 20-minute delay, the participant is asked to verbally recall as many words as they can from the list, in any order. | Delayed recall and retention score |
|  | Rey-Osterrieth Complex Figure Task (ROCF) [6,7] | The ROCF recall condition involves participants reproducing a complicated line drawing by drawing the image freehand from memory after a 20-min delay period. | Delayed recall score |
| Visuospatial Function | Rey-Osterrieth Complex Figure Task (ROCF) [6,7] | The ROCF copy condition involves participants reproducing a complicated line drawing by copying the image freehand while referring to the drawing. | Total copy score |
| Executive Function | Trail-Making-Test [8] - *Part B* | The Trail-Making Test is a paper-pencil measure comprising 25 circles distributed over a sheet of paper. In Part B, the circles include both numbers (1-13) and letters (A-L) and participants are required to draw lines to connect the circles in the ascending order, as well as alternating between the numbers and letters (i.e., 1-A-2-B-3-C, etc). | Time taken (sec) |
|  | Clock Drawing Task (CLOX) [9] | Participants are required to draw an analog clock that reads a particular time, as instructed by the examiner. | Organizational score |
|  | Rey-Osterrieth Complex Figure Task (ROCF) [6,7] | The ROCF copy condition involves participants reproducing a complicated line drawing by copying the image freehand while referring to the drawing. While  doing so, participants use a series of colored markers. This allows the experimenter to preserve a record of the  order in which elements of the figure were reproduced. | Copy organizational score |
| Language | Boston Naming Test (BNT; 30-item) [10] | The BNT involves presenting participants with a picture of an item and the participant is required to name the item. Phonemic and/or semantic cues may be required to assist the participant in naming the picture. | Total correct with no cue or with semantic cue |
|  | Controlled Oral Word Association Test (COWAT) [11] | The COWAT requires participants to produce as many words as they can that begin with a given letter (F, A, and S) within a 1-min time period.  Participants are instructed to exclude proper nouns, and similar words that start with the same sound but have a different suffix.  Participants are then instructed to generate as many animals as they can within a 1-min. time period. | Total words beginning with ‘F’, ‘A’, and ‘S’, as well as animals generated |

**References**

[1] Wechsler D. WAIS-III administration and scoring manual. The Psychological Corporation, San Antonio, Texas. 1997.

[2] Reitan RM, Wolfson D. The Halstead-Reitan neuropsychological test battery: theory and clinical interpretation: Reitan Neuropsychology; 1985.

[3] Westerman R, Darby DG, Maruff P, Collie A. Computer-assisted cognitive function assessment of pilots. ADF Health. 2001;2:29-36.

[4] Brandt J. The Hopkins Verbal Learning Test: Development of a new memory test with six equivalent forms. Clin Neuropsychol. 1991;5:125-42.

[5] Benedict RH, Schretlen D, Groninger L, Brandt J. Hopkins Verbal Learning Test–Revised: Normative data and analysis of inter-form and test-retest reliability. Clin Neuropsychol. 1998;12:43-55.

[6] Osterrieth P. Le test de copie d'une figure complex: contribution á l'étude de la perception et de la mémoire [The Complex Figure Test: contribution to the study of perception and memory]. Arch Psychol. 1944;28:1021-34.

[7] Rey A. L'examen psychologique dans les cas d'encéphalopathie traumatique (les problems). [The psychological examination in cases of traumatic encephalopathy (problems)]. Archives de Psychologie. 1941.

[8] Reitan RM, Wolfson D. The Halstead-Reitan neuropsychological test battery: theory and clinical interpretation: Reitan Neuropsychology; 1985.

[9] Royall DR, Cordes JA, Polk M. CLOX: an executive clock drawing task. J Neurol Neurosurg Psychiatry. 1998;64:588-94.

[10] Goodglass H, Kaplan E, Weintraub S. Boston Naming Test: Lea & Febiger, Philadelphia, PA; 1983.

[11] Benton AL, Hamsher KdeS, Sivan AB. Multilingual aphasia examination: AJA associates; 1994.
